# A cohort study among 402 patients with penile cancer in Maranhão, Northeast Brazil with the highest worldwide incidence

**DOI:** 10.1186/s13104-020-05283-z

**Published:** 2020-09-18

**Authors:** Ciro Bezerra Vieira, Antonio Teixeira-Júnior, Laisson Feitoza, Jaqueline Pinho, José Calixto, Francisco Sérgio Moura Silva do Nascimento, Marcos Adriano Garcia Campos, Joyce Lages, Antonio Machado Alencar Junior, Fernando Soares, Isabela Cunha, Gyl Eanes Barros Silva

**Affiliations:** 1 University Hospital of Federal University of Maranhão (UFMA), Barão de Itapari Street, Centro, São Luís, Brazil; 2 Postgraduate Program in Adult Health (PPGSAD), Federal University of Maranhão (UFMA), São Luis, MA Brazil; 3 Department of Radiology, University Clinic Hospital of Estadual University of Campinas, Campinas, Brazil; 4 Laboratory of Immunofluorescence and Electron Microscopy (LIME), Presidente Dutra University Hospital (HUUFMA), São Luís, MA Brazil; 5 Department of Medicine II, Federal University of Maranhão (UFMA), São Luís, MA Brazil; 6 Department of Pathology, School of Medicine, University of São Paulo, São Luís, Brazil; 7 Doctor at the Antônio Prudente Foundation, São Paulo, Brazil; 8 Department of Pathology, Ribeirão Preto Medical of School, University of São Paulo (USP), Bandeirantes Avenue, Monte Alegre, Ribeirão Preto, 14049-900 Brazil

**Keywords:** Carcinoma, Penile neoplasms, Papillomavirus infections, Squamous cell carcinoma

## Abstract

**Objective:**

Maranhão State—Northeast Brazil–has the world’s highest incidence of penile cancer. This study describes the epidemiological, histopathological and clinical profile of patients stricken across that Brazilian state. The study is aimed at providing new data on neoplasia.

**Data description:**

402 men stricken with penile cancer were studied from January 2004 to December 2018. A retrospective stage was developed with collection of physical and electronic records. A prospective stage was performed with collection of clinical and epidemiological information through a questionnaire. The surgical material was looked into by a uropathologist, and the lesions were evaluated for macroscopic characteristics and various microscopic parameters. Three articles using this data have already been published.

## Objective

The incidence of penile cancer is low in developed countries; however, this neoplasm is still a problem in developing countries, reaching alarming levels [1]. The main risk factors are strongly related to socioeconomic conditions, genital hygiene habits, characteristics related to the foreskin and human papillomavirus (HPV) infection [2, 3]. The state of Maranhão—Northeastern Brazil–has the highest reported incidence of cancer of the penis (6.1 cases/100,000 ASR) and this fact is due to the high prevalence of risk factors in this region [4]. Maranhão is a poor state with highest prevalence of HPV infection in the country and has high rates of phimosis among the male population [5, 6]. Penile cancer is a poorly studied topic compared to other more prevalent neoplasms, and few studies have been published on the population of the state of Maranhão. Thus, it is expected that the description of the characteristics of this population will contribute to the prevention and management of penile neoplasia, particularly with regard to the understanding of related risk factors with new information. These data sets are useful for comparing this population with that in other regions of the world and the significant number of cases analyzed also gives the study an important statistical significance. Few advances have been made in the management of penile neoplasia in recent years, and this study also aims to provide theoretical ground for further research of the diagnosis and treatment of this condition. Part of the data obtained in this study was used in three previously published studies [4, 5, 7], addressing the epidemiological and histopathological profile of penile cancer in Maranhão, as well as immunohistochemical analyzes related to the presence of HPV and of the p16INK4a protein in this sample.

## Data description

This is a retrospective and prospective cohort study carried out in the state of Maranhão—Northeastern Brazil–from January 2004 to December 2018. The total population studied comprised 402 men with penile cancer treated by spontaneous demand at the Urology ward of two public hospitals within the state’s capital city: University Hospital of the Federal University of Maranhão and Aldenora Bello Cancer Hospital. There were no exclusion criteria. The study variables included epidemiological, clinical and surgical characteristics of the participants and histopathological aspects of the penis cancer samples (see Table 1). The criterion used to classify genital hygiene as poor/moderate was less than one genital wash per day or the presence of phimosis [8]. The tumor subtype was described according to the revised and updated classification of penile carcinomas of the World Health Organization (2016) [9]. The histological grade of the tumor was described according to the classification by Velasquez et al. [10] and the staging was performed according to the classification of the TNM, 8th ed., 2017. Data collection was performed in two stages. First, all data corresponding to the period from January 2004 to December 2014 was collected retrospectively through physical and electronic records and amounted to 286 patients (see Table 1). From January 2015 to December 2018, the epidemiological and clinical data of 116 patients were prospectively collected through a questionnaire prepared by the research team (see Table 1); the questionnaire was applied by a single researcher directly to patients during outpatient consultations or during the period of hospital stay for surgical procedures. The surgical materials extracted from each patient were sent to the Laboratory of Immunofluorescence and Electron Microscopy at the University Hospital of the Federal University of Maranhão (LIME–HUUFMA). The lesions were photographed and those that were not included in paraffin were either completely embedded in paraffin or made at least 40 paraffin blocks for rather advanced lesions. The histopathological material was examined by a single experienced uropathologist (GEBS) for macroscopic characteristics and the various microscopic parameters. Some samples of paraffin tumor tissue and fresh tissue were selected for immunohistochemistry and analysis of the polymerase chain reaction (PCR), respectively. Histological identification and molecular analysis of HPV, as well as immunohistochemistry for expression of p16INK4a were performed according to the description contained in the study by Martins VA et al. [7]. The population data obtained in the first stage of the study were used to calculate the age-standardized incidence rate (ASR) of penile cancer in Maranhão [4] with the very same population. Categorical variables were presented by means of frequencies and percentages and numerical variables by means and standard deviations (mean ± SD). The data were analyzed using the Stata program, version 12.0. The association between categorical variables was analyzed using the Chi square test or Fisher’s exact test. The 95% confidence interval was calculated by logistic regression with a significance of p < 0.05.

**Table 1:** Overview of data files/data sets

Label	Name of data file/data set	File types (file extension)	Digital object identifier (DOI)
Data file 1	Retrospective profile of penile cancer in Maranhão (2004–2014)	.xlsx	Figshare (10.6084/m9.figshare.12470045.v4) [11]
Data file 2	Prospective profile of penile cancer in Maranhão (2016–2018)	.xlsx	Figshare (10.6084/m9.figshare.12470030.v4) [12]
Data file 3	P16^INK4a^ expression in patients with penile cancer	.xlsx	Figshare (10.6084/m9.figshare.12921941.v1) [13]

## Limitations

This study has some limitations: 1. The retrospective collection of part of the data came up against the difficulty in obtaining complete information about some variables which were sometimes not recorded or were recorded in an unreadable way within the physical records; however, the data obtained were sufficient to understand important aspects of penile cancer in Maranhão region. 2. Data collection through a questionnaire, in some cases, ran into the difficulty of understanding by some patients with low education; nevertheless, in five cases in which the collection was considered insufficient, patients were excluded from the study. 3. There was loss of monitoring or abandonment of treatment in many cases, probably due to the fact that they live in distant rural areas, with difficulty in accessing the hospitals participating in the study.

## Data Availability

The data described in this Data Note can be freely and openly accessed on the figshare dataset repositories in the following data records: [10.6084/m9.figshare.12470045.v4], [10.6084/m9.figshare.12470030.v4], [10.6084/m9.figshare.12921941.v1]. Please see Table 1 and references [11–13] for details and links to the data.

## Abbreviations

HPV: Human papillomavirus
STIs: Sexually transmitted infections
LIEM: Laboratory of Immunofluorescence and Electron Microscopy
